# Tissue Specific Promoters in Colorectal Cancer

**DOI:** 10.1155/2015/390161

**Published:** 2015-11-15

**Authors:** A. R. Rama, A. Aguilera, C. Melguizo, O. Caba, J. Prados

**Affiliations:** ^1^Department of Health Science, University of Jaen, Jaen, Spain; ^2^Institute of Biopathology and Regenerative Medicine (IBIMER), University of Granada, Armilla, 18100 Granada, Spain; ^3^Department of Human Anatomy and Embryology, School of Medicine, University of Granada, Granada, Spain; ^4^Biosanitary Institute of Granada (ibs GRANADA), SAS-Universidad de Granada, Granada, Spain

## Abstract

Colorectal carcinoma is the third most prevalent cancer in the world. In the most advanced stages, the use of chemotherapy induces a poor response and is usually accompanied by other tissue damage. Significant progress based on suicide gene therapy has demonstrated that it may potentiate the classical cytotoxic effects in colorectal cancer. The inconvenience still rests with the targeting and the specificity efficiency. The main target of gene therapy is to achieve an effective vehicle to hand over therapeutic genes safely into specific cells. One possibility is the use of tumor-specific promoters overexpressed in cancers. They could induce a specific expression of therapeutic genes in a given tumor, increasing their localized activity. Several promoters have been assayed into direct suicide genes to cancer cells. This review discusses the current status of specific tumor-promoters and their great potential in colorectal carcinoma treatment.

## 1. Background

Colorectal carcinoma (CRC) is the third most prevalent cancer in the world [1]. The main treatments, such as 5-fluorouracil (5-FU) alone or combined (FOLFOX and FOLFIRI), new angiogenesis inhibitors, and epidermal growth factor receptor inhibitors, induce a poor response in most advanced stages and are usually accompanied by other tissue damage [2]. Suicide gene therapy has been widely used in many studies* in vitro* and* in vivo*, demonstrating that it may potentiate the classical cytotoxic effects in some tumors [3], including colon cancer [4, 5]. However, gene therapy application in cancer patients has not yet successfully gained clinical significance. The inconvenience still rests with targeting and efficiency of the specificity.

**Figure 1:**
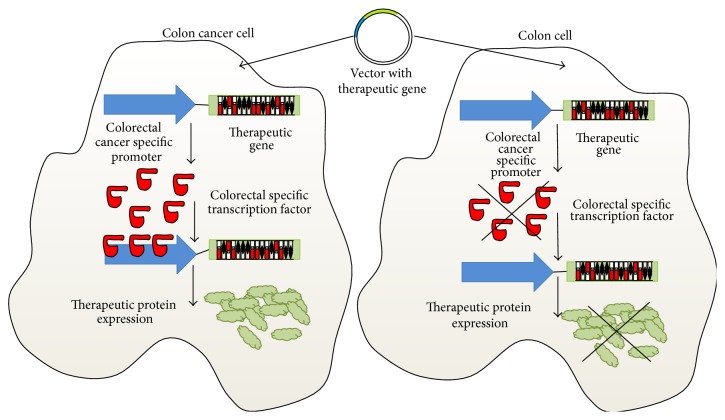
Schematic representation of a colon cancer specific promoter and the induction of therapeutic gene expression. High levels of specific transcription factors in colon cancer cells are able to induce therapeutic gene expression. By contrast, no expression of these specific transcription factors in normal colon cells avoids the transcription of the therapeutic gene.

For this purpose, it is necessary to express these genes into specific tumor cells. The main target of gene therapy is to achieve an effective vehicle to hand over therapeutic genes safely into specific cells. One possibility is the use of tumor-specific promoters, overexpressed in cancer cells. They could induce a specific expression of therapeutic genes in a type of tumor increasing their localized activity (Figure 1).

For instance, the* TTS* system (*TTF1* gene under the control of* hTERT* promoter and* hSPA1* promoter) shows a selective activity in lung cancer cells but not in other types of cancer or normal cells [6]. Other promoters employed in gene therapy are the *α fetoprotein* (*AFP*) promoter (for hepatic cancer) [7, 8] and the* erb2* promoter (for breast cancer) [9, 10]. It has been demonstrated that the hTERT promoter is able to direct the expression of the* PEA-15* gene, a tumor suppressor gene and inhibitor of cell growth and invasion [11–13], specifically to breast cancer cells, inducing growth suppression and inhibition. This decrease is also observed in the tumor growth of orthotopic animal models, as well as a prolongation of survival time [14]. Similar results have been found by Zhang et al. [15], who have proved the capacity of the tumor-specific promoter hTERT to drive the expression of the* apoptin* and* E1A* genes in prostate carcinoma cells and in mouse models. Apoptin, a protein derived from chicken anemia virus VP3 gene, is able to induce selective apoptosis in human tumor and transformed cells but shows little or no cytotoxic effect in many normal human cells [16, 17]. Several promoters such as carcinoembryonic antigen (CEA), cyclooxygenase-2 (COX-2) [18], human telomerase reverse transcriptase [19], and Urokinase-type plasminogen activator receptor (uPAR) [20] have been assayed to direct suicide genes into CRC cells.

In this review, we are going to show the current status of specific tumor-promoters and their great potential in CRC treatment.

## 2. Tumor-Promoters in Colorectal Carcinoma

### 2.1. CEA

**Figure 2:**
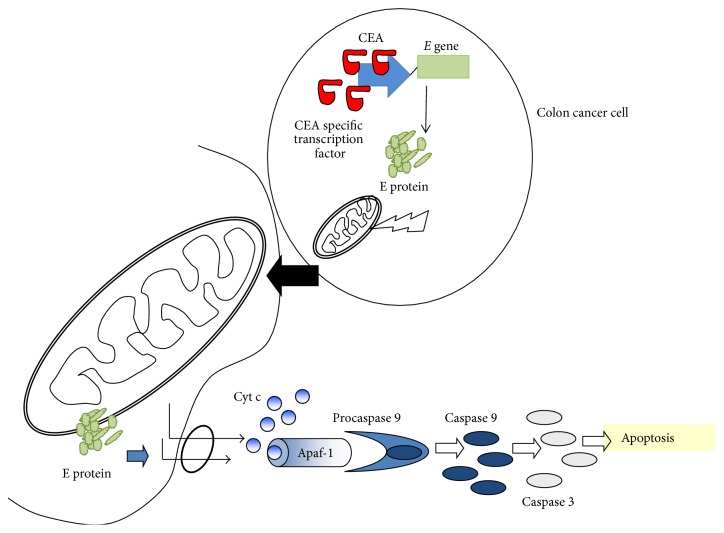
Antitumor effect of the *E* gene under CEA promoter. The high transcriptional activity of the CEA promoter in colon cancer cells leads to *E* gene expression which encodes a cytotoxic protein. The protein *E* targets mitochondria in colon cancer cells, disrupting their cristae and inducing apoptosis by release of cytochrome c and activation of caspases 9 and 3.

Carcinoma embryonic antigen (CEA) is an oncofetal tumor marker overexpressed in over 90% of colorectal cancer cells but not in normal colon cells [21–23]. High levels of serum CEA and high expressions of CEA mRNA have been detected in patients in the last stages of human colon carcinogenesis [24, 25]. CEA levels have been used for predicting the prognosis and monitoring recurrence and metastasis in patients with stage II CRC [26]. In fact, CEA showed clinical and pathological significance as prognostic markers in the diagnosis of colorectal cancer [26, 27], local recurrence, and overall survival after resection [28]. This elevated* CEA* promoter expression has also been shown in cancer cell lines versus nontumor cell lines [29, 30]. In response to this tumor specificity,* CEA* promoter has been studied to drive the expression of therapeutic genes to CEA positive cancer cells [18]. Zhang et al. [30] studied the efficiency of the double system cytosine deaminase (*CD*) and thymidine kinase (*TK*) targeted by* CEA* promoter in CEA positive human gastric cancer cell line (SGC7901) versus a CEA negative human adenocarcinoma cell line (HeLa), showing a greater growth inhibition in SGC7901 (89.8%) than in HeLa line cell (2%). Similar findings were revealed in the CEA positive human colon cancer cell line (LoVo). After 5 days of 5-FC treatment, HeLa cells transfected with CEA-*CD* were not sensitized by the cytotoxicity, whereas transfected LoVo cells showed a cell growth inhibition of 72.7% [31].* In vivo* studies demonstrated a similar effect in LoVo xenografts mice treated with the CEA-*CD* system [31] and in xenograft SGC7901 treatment with the double system CEA-*CD*-*TK* (46% tumor growth inhibition rate (TGIR) versus nontreated tumor control) [30]. Current study of Rama et al. [32] reveled the ability of the* CEA* promoter to direct *E* gene expression towards colon cancer cells, inducting a high cell growth inhibition in comparison to normal human colon cells (Figure 2). In addition,* in vivo* analyses of mice bearing subcutaneous MC-38 colon cancer cells showed a significant decrease in tumor volume and low level of Ki-67 in relation to untreated tumors.

### 2.2. Cox-2

Cyclooxygenase-2 (Cox-2) is an enzyme which participates together with COX-1 in the oxidation of arachidonic acid to prostaglandin, an essential promoting factor in carcinogenesis and development of tumors [33, 34]. Some studies have demonstrated that uses of inhibitors against Cox-2 suppress colon carcinogenesis [34, 35]. Cox-2 is associated to CRC [36], exhibiting expression in 93% of colon cancers and in 87% of rectal cancers [37], to polyps with high-grade [38, 39], to a higher TNM (tumor, node, metastasis) class, and to higher Dukes' stage [40]. In a study on 35 cases of CRC, 77% of them were Cox-2 positive and 43% showed location in the rectum and left side [41]. This overexpression has been associated with the reduced survival of CRC patients [42]. Furthermore, the recent study has shown higher values of expression in colon cancer (93%) than in rectal cancer (87%), associating this decrease of Cox-2 expression to decreased disease-specific survival and decreased disease-free survival in rectal cancer but not in colon cancer, suggesting the Cox-2 expression as a predictive clinical biomarker of rectal but not colon cancer [37].

This elevated* Cox-2* promoter expression has also been shown in cancer cell lines versus nontumor cancer cell lines [43–45]. Wang et al. [45] analyzed the transcriptional activity of Cox-2 promoter by the* luciferase* reporter gene in colorectal cancer cell lines and normal human intestinal epithelial cell lines. The results proved an increased luciferase activity in all colorectal cancer lines (a median of 83% of the three of them) relative to normal cells (12%). Based on this specific-tumor activity,* Cox-2* promoter has been used to target different genes to specific colon cancer cells [43, 45]. The system Cox-2-*TK* conferred ganciclovir sensitivity to LoVo tumor cells and 52.5 ± 1.2% inhibitory rates but did not affect normal cells [45]. Another similar study has used the Cox-2 promoter to drive the* 15-hydroxyprostaglandin dehydrogenase* (*15-PGDH*), a gene suppressed in the majority of cancers.* 15-PGDH* specific expression, under* Cox-2* control promoter in colon cancer cells, inhibited growth and migration of colon cells [43].* In vivo* studies demonstrated a similar effect in LoVo xenografts treated with Cox-2-*TK*, showing 59.4% inhibitory rates versus nontreated LoVo xenografts [45]. Thus, Kaliberova et al. [43] corroborated the effect of the system Cox-2-*15-PGDH* in LS174T xenografts, disclosing an inhibitory effect on tumor growth compared to nontreated xenografts.

### 2.3. A33

A33 is a transmembrane glycoprotein member of the immunoglobulin superfamily, present only in the small intestine and colon [20, 46] and is associated with the process of cell adhesion, cell trafficking, and intestinal immune response [47, 48]. A33 overexpression is related to several cancers such as primary and metastatic colorectal carcinomas (95%), diffuse gastric cancers (63%), intestinal-type gastric cancers (83%), and pancreatic cancers (50%) but has been undetected in normal epithelial tissue [49, 50]. However, the expression level of A33 is not correlated to the disease stage and the degree of histological differentiation [51].

Having established this specific expression of A33 in gastrointestinal cancer, several immunotherapy assays have manipulated different humanized A33 antibody fragments, targeting them as specific carriers of other molecules (immunoconjugates) for antitumor treatment [52–54]. Recently, Cafferata et al. [55] have used the A33 promoter in the design of a conditionally replicative adenovirus to specifically drive the essential early* E1A* gene into CRC cells. E1A is an oncoprotein with several anticancer activities such as decreasing tumorigenic potential, increasing inhibition of cell growth and promoting apoptosis [56, 57]. They showed A33 mRNA expression levels in different colorectal carcinoma cell lines, but not in normal colonic cells, breast cancer cell lines, hepatocellular carcinoma cell lines, fetal lung fibroblast cell lines, melanoma cell lines, and embryonic kidney cells. This was related to the activity of the* A33* promoter, essentially active only in human CRC cells whereas human mammary and melanoma cells showed strongly reduced activity. Subsequently, the adenovirus showed specific lytic activity in human colorectal carcinoma cell lines and a slight activity in hepatocellular carcinoma and melanoma cell lines. To improve this therapeutic effect, the A33-*E1A* adenovirus was combined with 5-FU administration, exhibiting an enhanced lytic effect of 5-FU colon cancer cell lines compared with the 5-FU treatment alone.* In vivo*, the adenovirus was effective in inhibiting tumor growth in 100% of LoVo xenografts; treated mice survived significantly longer than the control group. However, no evidence was observed in melanoma xenografts. Also, liver metastasis was studied, displaying absence of metastatic nodules (10/11 mice injected with A33-*E1A* adenovirus) and strongly reduced metastatic areas (1/11). Nonetheless, adding 5-FU in combination with the A33-*E1A* adenovirus did not significantly improve the tumor growth inhibitory effect observed with A33-*E1A* adenovirus alone.

### 2.4. TERT

Telomere/telomerase interplay has a prominent role in the preservation of genetic chromosome stability and its failure is involved in carcinogenesis [58]. Human telomerase has two subunits: a template RNA component (hTR) and a catalytic subunit called the human telomerase reverse transcriptase (hTERT) [59]. The expression of hTR subunit is expressed in all types of human cells and serves as a template for telomere synthesis; however, hTERT is expressed in cells with high telomerase activity, as tumor cells, but it is not expressed in normal tissues [59–62]. Telomerase is highly active in 90% of malignant tumors [63]. CRC patients with increased levels of hTERT mRNA have been correlated with tumor stage, histological grade, and significantly worse survival than CRC patients with low hTERT levels [58, 64].

Higashi et al. [19] confirmed, using EGFP as reporter gene, the high activity of the hTERT promoter in several tumor cell lines of human esophageal cancer and mouse colon adenocarcinoma, but they did not find activity in normal human fibroblasts. The hTERT promoter has been used to direct the therapeutic genes expression in cancer showing a great tumor-specific capacity [62, 65–67].

Yang et al. [68] used an adenovirus based on the hTERT promoter to deliver both* apoptin* gene and* E1A* gene into CRC cells. This adenovirus induced 70–75% of cell growth inhibition in CRC cells, showing 32.3% and 31.5% levels of apoptosis and necrosis, respectively. Conversely, no effect was observed in transfected human gastric epithelium. In concordance with these results, the* in vivo* experiments with mouse models of CRC proved that this adenovirus provoked a slower tumor growth, increased the median survival time, and reduced the number of metastatic lung nodules with respect to the nontreated CRC mice. Higashi et al. [19] utilized the hTERT promoter to direct in a specific way the expression of two genes,* interleukin-18* (*IL-18*) and* TK*, to murine colorectal cancer cells. IL-18 is a proinflammatory cytokine that activates the cytotoxicity of CD8^+^ T, CD4^+^ T, and NK cells [69, 70]. The mentioned cells were sensitive to ganciclovir and showed high levels of IL-18 secretion. These cells were injected into mice in order to generate colorectal cancer tumors in them. After treating them with ganciclovir, the mentioned tumors were totally eliminated, whereas in the control groups the tumor growth was progressive. Besides, a rise of CD8^+^ T and CD4^+^ T cells in the tumor zone was observed, indicative of tumor-specific acquired immunity.

### 2.5. uPAR

*Urokinase-type plasminogen activator receptor* (*uPAR*) gene codes a serine protease that catalyzes the transformation of the inert zymogen plasminogen into plasmin [20, 71].* uPAR* gene is upregulated by the activated RAS signaling pathway, the main signaling pathway activated in colon cancer [72]. The components of the* uPAR* system are overexpressed in diverse human tumors, such as pancreatic, hepatic, breast, and especially gastrointestinal cancers [73–76]. Tumor specific binding of activator protein (AP-1) to* uPAR* promoter has been detected in ~40% CRC patients, and 39.8% of them showed this tumor specific binding in the resected tumors in contrast to low or absent binding in corresponding normal mucosa [76] demonstrating the tumor specific activity of* uPAR* in CRC and not in normal tissue. High uPAR protein levels have been correlated with poor 5-year survival in colon cancer patients [50] and increased invasive capacity of tumor cells [77].

Teimoori-Toolabi et al. [78] proved the specific activity of the* uPAR* promoter in colon and colorectal cancer cell lines. Using the* LacZ* gene reporter under the control of the* uPAR* promoter, they observed beta-gal expression in human colorectal carcinoma (HTC116) and in colon cancer cells (SW480), but not in normal colon cells and nontransformed human umbilical vein endothelial cells. Afterward, they used* uPAR* promoter to deliver* TK* gene in SW480 and HCT116 cells. The growth of these cells with ganciclovir was significantly decreased.

### 2.6. FGF18

*Fibroblast growth factor 18* (*FGF18*) is a crucial mitogen in embryonic limb development [79] with a significant participation in the development of cartilage and bone [80, 81]. Its overexpression has been associated to different types of cancer, especially CRC [82, 83].* FGF18* is downstream of Wnt pathways and is highly active in CRC [56, 82, 84]. In a study with 38 CRC and their respective normal mucosa, 34 out of 38 CRC exhibited greater FGF18 mRNA levels than the normal mucosa. Moreover, this overexpression was associated with colon carcinogenesis from adenoma to carcinoma [84], suggesting* FGF18* as a novel marker for early detection of colorectal tumors [82].

Teimoori-Toolabi et al. [85] researched the* FGF18* promoter activity in SW480, HCT116, human normal colon cells, and umbilical vein endothelial cells. All cells were transiently transfected with a plasmid with* LacZ* gene reported under* FGF18* promoter. Beta-gal staining showed a higher expression in SW480 (5%) and HCT116 (10%) than in human normal colon cells and umbilical vein endothelial cells (0%). After demonstrating the tumor specific activity of* FGF18* promoter, this was used in a new plasmid to deliver* TK* gene to cancer cells. A significantly decreased growth was shown in SW480 and HCT116 cells after ganciclovir treatment.

### 2.7. KDR

The endothelial cell type-specific tyrosine kinase domain-containing receptor (KDR) is a receptor for the vascular endothelial growth factor (VEGF), playing an essential role in endothelial cell growth and development [86]. KDR expression has been detected in a variety of cancer cells and neogenetic vascular endothelial cells of the neoplasm but has not been detected in normal cells [86–89]. Currently, in a study with 110 CRC patients, single nucleotide polymorphisms (SNP) of* KDR* were correlated with microvessel density and overall survival [89]. Hansen et al. [90] also linked SNP of* KDR* with a reduced recurrence risk, this association being higher in CRC patients receiving chemotherapy.

The specific activity of the* KDR* promoter to deliver both* TK* and* CD* genes (KDR/CD-TK) in colon cancer cells has been studied. CD/TK mRNA levels were detected in SW480 and SW620 cells (KDR positive human colon adenocarcinoma) exhibiting both high sensibility to the prodrugs 5-FC and ganciclovir. However, none of these results were observed in LS174T cells (KDR negative human colon carcinoma) [91, 92].

## 3. Conclusions

Tissue-specific promoters are able to improve gene delivery to tumor tissue, reducing at the same time the effect on healthy tissues and increasing the efficacy against cancer cells. Currently, a great amount of tumor-specific promoters are known and several* in vivo* and* in vitro* assays have revealed their specific activity in CRC, as well as their potential use. However, more assays will be needed in order to demonstrate and enhance their efficacy. One possibility is the use of enhancers, whose assays have proven to increase the transcriptional activity of these promoters. The use of tissue-specific promoters to deliver the expression of suicide genes for the selective killing of tumors may be a novel strategy for cancer treatment.
